# The profile of older adults seeking chiropractic care: a secondary analysis

**DOI:** 10.1186/s12877-021-02218-6

**Published:** 2021-04-23

**Authors:** Katie de Luca, Sheilah Hogg-Johnson, Martha Funabashi, Silvano Mior, Simon D. French

**Affiliations:** 1 Department of Chiropractic, Faculty of Medicine, Health and Human Sciences, Macquarie University, Sydney, Australia; 2 Department of Research, Canadian Memorial Chiropractic College, Toronto, Canada; 3 Centre for Disability Prevention and Rehabilitation, Ontario Tech University and Canadian Memorial Chiropractic College, Toronto, Canada

**Keywords:** Back pain, Low back pain, Neck pain, Musculoskeletal conditions, Aging, Elderly, Health services, Chiropractic, Observational study

## Abstract

**Background:**

Musculoskeletal conditions are the primary reason older adults seek general medical care, resulting in older adults as the highest consumers of health care services. While there is high use of chiropractic care by older adults, there is no recent, specific data on why older adults seek chiropractic care and how chiropractors manage conditions. Therefore, the purpose of this study was to describe the demographic characteristics of older adults seeking chiropractic care, and to report problems diagnosed by chiropractors and the treatment provided to older adults who seek chiropractic care.

**Methods:**

A secondary data analysis from two, large cross-sectional observational studies conducted in Australia (COAST) and Canada (O-COAST). Patient encounter and diagnoses were classified using the International Classification of Primary Care, 2nd edition (ICPC-2), using the Australian ICPC-2 PLUS general practice terminology and the ICPC-2 PLUS Chiro terminology. Descriptive statistics were used to summarize chiropractor, patient and encounter characteristics. Encounter and patient characteristics were compared between younger (< 65 years old) and older (≥65 years old) adults using χ^2^ tests or t-tests, accounting for the clustering of patients and encounters within chiropractors.

**Results:**

A total of 6781 chiropractor–adult patient encounters were recorded. Of these, 1067 encounters were for persons aged > 65 years (16%), from 897 unique older patients. The most common diagnosis within older adult encounters was a back problem (56%), followed by neck problems (10%). Soft tissue techniques were most frequently used for older patients (85 in every 100 encounters) and in 29 of every 100 encounters, chiropractors recommended exercise to older patients as a part of their treatment.

**Conclusions:**

From 6781 chiropractor–adult patient encounters across two countries, one in seven adult chiropractic patients were > 65 years. Of these, nearly 60% presented with a back problem, with neck pain and lower limb problems the next most common presentation to chiropractors. Musculoskeletal conditions have a significant burden in terms of disability in older adults and are the most commonly treated conditions in chiropractic practice. Future research should explore the clinical course of back pain in older patients seeking chiropractic care and compare the provision of care to older adults across healthcare professions.

## Background

Ageing of the population is a global phenomenon and high-income countries, such as Australia and Canada, are currently experiencing rapid growth of older age groups [1]. It is predicted that in both Australia and Canada, 22% of the population will be aged over 65 years by 2042 [2, 3] and globally there will be a threefold increase in persons aged over 80 years by 2050 [1]. The prevalence and socioeconomic burden of musculoskeletal conditions, and resulting disability, increases with age [4]. In Australia, musculoskeletal conditions contribute to 9% of total disability adjusted life years for those aged over 65 years [5]. Globally musculoskeletal conditions contribute to 8% of total disease burden in those aged over 60 years [6, 7]. Low back pain in older adults is more likely to be moderate to severe, and is more likely to be incapacitating, when compared to younger adults [8]. Older adults with low back pain, with or without accompanying leg pain, are twice more likely to face increased difficulty in lifting, walking or bathing themselves [9, 10] and lifting objects, housework, climbing stairs and walking, than older patients without pain [11]. Musculoskeletal conditions have a negative effect on an older person’s health and quality of life [12]; decreasing mobility, reducing social participation, increasing isolation and creating feelings of helplessness and frustration [13]. Economically in 2018, musculoskeletal conditions cost the Australian health system $9.3 billion, while in Canada, older adults account for approximately 45% of provincial health care expenditures [14].

Chiropractic is a health profession concerned with the diagnosis, treatment and prevention of mechanical disorders of the musculoskeletal system, and the effects of these disorders on the function of the nervous system and general health [15]. Globally, the median annual utilisation of chiropractic services is 9% [16], which increases to 15% in older adults [17]. In Australia, 73% of chiropractors report regularly treating adults aged older than 65 years [18] and, of patients who present to a chiropractor, 12% are aged older than 65 years [19]. In Canada, there is a higher proportion of older chiropractic patients and 19% of patients are older than 65 years [20]. While there is a high use of chiropractic care by older adults, and there is a high proportion of older adults as chiropractic patients, information about why older people seek chiropractic care and what care chiropractors provide are either nearly 20 years old [21, 22], or limited as they were collected using administrative databases from the United States [17]. In terms of treatment, consensus, evidence-based statements regarding an appropriate approach to chiropractic care in older adults [23], which include the safety of manipulation and advice on exercise for older patients [24], exist. A limitation is however that best practice recommendations must rely heavily on multidisciplinary, expert opinion due to sparse scientific evidence for the management of musculoskeletal conditions in this special population.

The Chiropractic Observation and Analysis STudy (COAST) was a cross-sectional, observational study, that described 4464 clinical encounters from chiropractors in Victoria, Australia [25]. Subsequently, O-COAST collected similar data on 3523 clinical encounters from chiropractors in Ontario, Canada [20]. As no recent studies have specifically analysed older patient data, the purpose of this study was describe demographic characteristics of the older chiropractic patient, report the extent of problems diagnosed in this population and report treatment provided by the chiropractor to older adults. We also compared problems diagnosed and the provision of treatment between younger (< 65 years) and older (≥65 years) adult chiropractic patients.

## Methods

### COAST & O-COAST

This study was a secondary data analysis from the COAST and O-COAST studies. The methods of COAST and O-COAST have been previously reported [20, 25]. Briefly, in COAST, from 1298 registered chiropractors in Victoria, 180 randomly selected chiropractors were invited to participate in a cross-sectional observational study of chiropractic practice in Victoria, Australia. Of 156 chiropractors who were eligible to participate, 72 chiropractors agreed, and 52 participated in the study between December 2010 and September 2012 (33% response rate). In O-COAST, from the 3978 chiropractors registered with the College of Chiropractors of Ontario in 2014, 135 randomly selected chiropractors were invited to participate. Of 120 chiropractors who were eligible to participate, 43 agreed, and 42 participated in the study between August 2014 and November 2015 (35% response rate). Sociodemographic and clinical practice data from the participating chiropractors in both studies were collected. For this secondary analysis, encounter data were included if the patient age was recorded as ≥18 years of age.

### Patient encounters

In both studies, chiropractors were asked to record patient encounter data by hand on standardized paper encounter recording forms for 100 consecutive encounters, with items in free text or check box format [25]. In both studies, the recording forms were piloted by five chiropractors with varying practice styles who each collected data on 10 consecutive patients. Chiropractors recorded information for each encounter included patient date of birth, postal code, sex, height, weight, date of encounter and up to three patient reasons for encounter. Chiropractor diagnoses plus the techniques and care provided for each diagnosis, whether the patient was referred by the chiropractor to another healthcare practitioner, whether imaging was ordered or performed, and how payment for the visit was made was recorded. Chiropractors also recorded information about the patients’ health characteristics such as comorbidities, diet, physical activity, smoking, alcohol consumption, quality of life, and general health status. This information was collected by the chiropractor by asking the patient standardized health assessment questions.

Reasons for encounter, diagnoses and comorbidities were classified by a trained coder at data entry using the *International Classification of Primary Care,* 2nd edition (ICPC-2), using the Australian ICPC-2 PLUS general practice terminology [26] and the ICPC-2 PLUS Chiro terminology [27]. ICPC-2 uses a three character alpha-numeric code to classify symptoms/complaints, problems/diagnoses or processes of care (called a rubric), and the ICPC-2 PLUS uses a further three digit code digit code to align the specific problem/diagnosis, or type of care with the most appropriate ICPC-2 rubric. As such the final three digits of the six-character ICPC-2 PLUS code simply serve to identify the specific term within the rubric and do not have any other meaning. For example, in ICPC-2 PLUS there are 11 neck-related terms in the L01 rubric that are regularly used by GPs in Australia to ‘Neck Symptom or Complaint’.

### Comorbidities

Comorbidities were reported by chiropractors within a free text box format. Comorbidities were then grouped under the following labels, being consistent with the ICPC symptom-based coding system where possible: cardiovascular disease (e.g., anaemia, blood dyscrasias, high blood pressure, hyperlipidaemia, stenosis); cancer; diabetes; gastrointestinal complaints (e.g., Crohn’s disease, cirrhosis, colitis, diverticulitis, hepatitis); genitourinary conditions (e.g., polycystic ovarian syndrome, prostatic hypertrophy, recurrent urinary tract infections); musculoskeletal conditions (e.g., fibromyalgia, osteoarthritis, osteoporosis, polymyalgia rheumatica, rheumatoid arthritis); neurological disease (e.g., Huntington’s disease, motor neuron disease); psychological conditions (e.g., depression, anxiety, psychiatric disease); respiratory conditions (e.g., asthma, bronchitis, chronic obstructive pulmonary disease, emphysema, pneumonia); and other complaints (e.g., auto-immune diseases, blindness, chronic skin conditions, glaucoma, sleep disorders).

Our study conforms to the appropriate reporting guidelines for observational studies (cross-sectional studies) in accordance with the STROBE (Strengthening the Reporting of OBservational studies in Epidemiology) guidelines [28]. COAST was approved by the University of Melbourne Human Research Ethics Committee (HREC: 0931651) and O-COAST approved by the Canadian Memorial Chiropractic College (REB: 1404X03) and Queen’s University (REB: 6012853) ethics boards.

### Statistical analysis

**Fig. 1:**
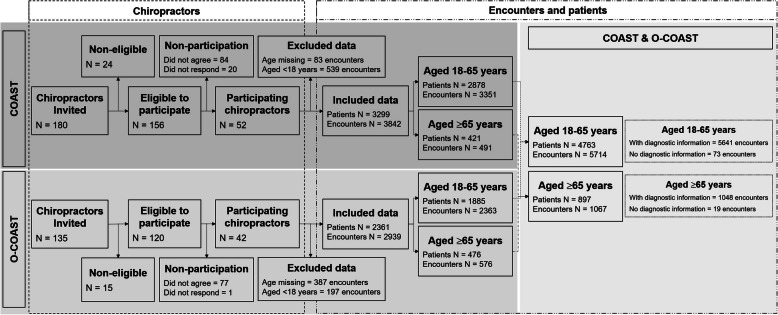
O-/COAST Older BMC Geriatrics flow chart

A flow chart of chiropractor, patient and patient encounter eligibility, inclusion and analysis was tracked using a flow diagram (Fig. 1). Descriptive statistics (frequency and percentages for categorical variables; mean, standard deviation and range for continuous variables) were used to summarize chiropractor, patient and encounter characteristics. When data were missing, they were not included in the computations of the statistics. The number of valid observations used for computations is reported in the tables. For some variables included (e.g., comorbidities, techniques used), it is not possible to distinguish data missing because it was not reported versus it was not applicable. Unique individual patients with multiple encounters were identified using the identifier assigned to the chiropractor combined with patient date of birth, postal code and sex. Chiropractor characteristics (sex, age, years in practice, years since graduation, country of education, number of adult encounters reported, number of adult patients reported, and percentage of patient ≥65 years old) were compared between COAST and O-COAST using χ^2^ tests or t-tests as appropriate. Encounter and patient characteristics were compared between younger (< 65 years old) and older (≥65 years old) adults using χ^2^ tests or t-tests, accounting for the clustering of patients and encounters within chiropractors by using survey estimator procedures with chiropractor as the primary sampling unit. All analyses were conducted using Stata version 10 (StataCorp. 2007. Stata Statistical Software: Release 10. College Station, TX: StataCorp LP) and SAS software (Copyright© 2018 SAS Institute Inc. SAS and all other SAS Institute Inc. product or service names are registered trademarks or trademarks of SAS Institute Inc., Cary, NC, USA).

## Results

### COAST and O-COAST chiropractors

A total of 52 chiropractors and 39 chiropractors participated in COAST and O-COAST, respectively. Patient age was not provided by two O-COAST chiropractors and data from one O-COAST chiropractor were excluded due to questionable responses on the encounter data (Fig. 1). In all tables, where the number of observations used to construct the statistic is different from the number of observations reported in the column heading, the number of valid observations is reported in the row heading. Table 1 details the characteristics of participating chiropractors from both studies; 70% of chiropractors were male, with a mean age of 43.1 years and were 16.1 (±9.7) years in practice. Chiropractors’ characteristics such as sex, age, years in practice and years since graduation were similar between the two studies. In both studies, 85% of chiropractors received their chiropractic education in their own country.

**Table 1:** Description of chiropractors for the total combined sample, and for COAST and O-COAST chiropractors^c^

	Total combined sample (***n*** = 91)	COAST Chiropractors (***n*** = 52)	O-COAST Chiropractors (***n*** = 39)	Comparison^**a**^ between COAST and O-COAST
Female (%)	27 (29.7%)	14 (26.9%)	13 (33.3%)	χ^2^ = 0.4, df = 1, *p* = 0.5
Mean age in years (range; SD)	43.1 (24–71; 10.3)	42.3 (24–64; 9.3)	44.1 (25–71; 11.7)	t = −0.84, df = 89, *p* = 0.4
Mean years in practice^b^ (range, SD) (*N* = 89)	16.1 (1–45; 9.7)	16.3 (1–39; 8.5)	15.8 (2–45; 11.2)	t = 0.82, df = 88, *p* = 0.8
Years since graduation (range, SD)	17.1 (1–45; 9.8)	16.9 (1–40; 8.7)	17.4 (3–45; 11.3)	t = 0.8, df = 89, p = 0.8
Less than 10 years	25 (27.5%)	13 (25.0%)	12 (30.8%)	χ^2^ = 1.0, df = 2, *p* = 0.6
Between 10 to 20 years	38 (41.8%)	24 (46.2%)	14 (35.9%)	
Greater than 20 years	28 (30.8%)	15 (28.9%)	13 (33.3%)	
Country of chiropractic education:				
Australia	44 (48.4%)	44 (84.6%)	–	
Canada	34 (37.4%)	1 (1.9%)	33 (84.6%)	χ^2^ = 77.2, df = 4, < 0.0001
United States	10 (11.0%)	4 (7.7%)	6 (15.4%)	
United Kingdom	1 (1.1%)	1 (1.9%)	–	
New Zealand	2 (2.2%)	2 (3.9%)	–	
Mean number of adult encounters (range; SD)	74.5 (7–105; 26.0)	73.9 (7–104; 26.6)	75.4 (13–105; 25.3)	t = −0.3, df = 89, p = 0.8
Mean number of adult patients (range; SD)	62.2 (5–99; 24.8)	63.4 (7–95; 23.8)	60.5 (5–99; 26.3)	t = 0.6, df = 89, p = 0.6
% Patients ≥65 years Mean (range; SD)	15.8 (0–80; 11.2)	12.5 (0–35; 7.9)	20.2 (1.25–80.0; 13.3)	t = −3.4, df = 89, *p* = 0.002

^a^Pearson χ^2^ test for categorical variables, t-test for means

^b^ For mean years in practice, *N* = 89. For all other variables data from all 91 chiropractors was used for analysis

^c^In both COAST and O-COAST studies, participating chiropractors were representative of respective, national chiropractors in terms of age, gender, location of practice and time since graduation

### Older adult patient demographics and patient encounters

From the 4464 COAST and 3523 O-COAST clinical encounters, 3842 and 2939 encounters were identified for adult patients (≥18 years), respectively. Therefore, in total there were 6781 chiropractor-adult patient encounters included in this analysis. From COAST, 421 patients were aged ≥65 years, with chiropractors providing information on 491 older adult patient encounters. From O-COAST, 476 patients were aged ≥65 years, with chiropractors providing information on 576 older adult patient encounters. Although COAST chiropractors had a smaller proportion of patients aged ≥65 years old (13%) in comparison to O-COAST chiropractors (20%), in the combined dataset, patients aged ≥65 years comprised 16% of all chiropractic adult patients (*n* = 897) and accounted for 16% of all adult patient encounters (*n* = 1067) (Fig. 1).

Table 2 reports sociodemographic characteristics of patients aged < 65 years and ≥ 65 years old. Most older adult patients were females (59%), had one chiropractic encounter during the data collection period (87%), were returning patients (97%) and were aged between 65 and 74 years old (69%). Three quarters of older patients were retired (77%). Overall, older adult patients presented with a greater number of comorbidities than younger adult patients, with the majority of older patients having cardiovascular disease (24%) and musculoskeletal problems (14%). In comparison to younger adults, older adult patients were less physically active (χ^2^ = 62.5, *p* < 0.0001) and were more often referred from a general/family practitioner (χ^2^ = 4.0, *p* = 0.046).

**Table 2:** Sociodemographic characteristics of total combined adult patient sample, for patients aged ≥65 and < 65 years at the patient level

	Total combined adult patient sample (*n* = 5660)	≥65 years old patients (*n* = 897)	< 65 years old patients (*n* = 4763)	Comparison^a^ between ≥65 years old and < 65 years old
Source				
COAST	3299 (58.3%)	421 (46.9%)	2878 (60.4%)	χ^2^ = 17.7 df = 1 *p* < 0.0001
O-COAST	2361 (41.7%)	476 (53.1%)	1885 (39.6%)	
Number of encounters per patient				
1	4898 (86.5%)	777 (86.6%)	4121 (86.5%)	χ^2^ = 1.1 df = 2 *p* = 0.59
2	556 (9.8%)	93 (10.4%)	463 (9.7%)	
3+	206 (3.7%)	27 (3.0%)	179 (3.8%)	
New Patient (*n* = 4845)				
Yes	318 (6.6%)	26 (3.3%)	292 (7.2%)	χ^2^ = 22.6 df = 1 *p* < 0.0001
No	4527 (93.4%)	771 (96.7%)	3756 (92.8%)	
Sex (*n* = 5554)				
Male	2320 (41.8%)	367 (41.4%)	1953 (41.8%)	χ^2^ = 0.08 df = 1 *p* = 0.77
Female	3234 (58.2%)	519 (58.6%)	2715 (58.2%)	
Age				
18–24	334 (5.9%)	–	334 (7.0%)	
25–54	3386 (59.8%)	–	3386 (71.1%)	
55–64	1043 (18.4%)	–	1043 (21.9%)	
65–74	619 (10.9%)	619 (69.0%)	–	
75–84	232 (4.1%)	232 (25.9%)	–	
85+	46 (0.8%)	46 (5.1%)	–	
BMI (*n* = 5457)				
Mean (range; SD)	26.9 (14.6–52.3; 5.2)	27.2 (16.7–48.9; 5.1)	26.9 (14.6–52.3; 5.2)	t = 1.33 df = 87 *p* = 0.19
Comorbidities				
Cancer	63 (1.1%)	28 (3.1%)	35 (0.7%)	χ^2^ = 70.9 df = 1 *p* < 0.0001
Cardiovascular	493 (8.7%)	217 (24.2%)	276 (5.8%)	χ^2^ = 334.1 df = 1 *p* < 0.0001
Diabetes	171 (3.0%)	62 (6.9%)	109 (2.3%)	χ^2^ = 37.6 df = 1 *p* < 0.0001
Gastrointestinal	92 (1.6%)	24 (2.7%)	68 (2.7%)	χ^2^ = 11.6 df = 1 *p* = 0.0006
Genitourinary	40 (0.7%)	9 (1.0%)	31 (0.7%)	χ^2^ = 1.4 df = 1 *p* = 0.2
Musculoskeletal	288 (5.1%)	129 (14.4%)	159 (3.3%)	χ^2^ = 177.3 df = 1 *p* < 0.0001
Neurological	52 (0.9%)	22 (2.5%)	30 (0.6%)	χ^2^ = 19.4 df = 1 *p* < 0.0001
Psychological	129 (2.3%)	18 (2.0%)	111 (2.3%)	χ^2^ = 0.6 df = 1 *p* = 0.4
Respiratory	113 (2.0%)	27 (3.0%)	86 (1.8%)	χ^2^ = 5.8 df = 1 *p* = 0.02
Other	154 (2.7%)	28 (3.1%)	126 (2.7%)	χ^2^ = 0.5 df = 1 *p* = 0.5
# Comorbidity Categories				
0	4413 (78.0%)	484 (54.0%)	3929 (82.5%)	χ^2^ = 311.6 df = 5 *p* < 0.0001
1	791 (14.0%)	230 (25.6%)	561 (11.8%)	
2	291 (5.1%)	108 (12.0%)	183 (3.8%)	
3	99 (1.8%)	46 (5.1%)	53 (1.1%)	
4	33 (0.6%)	14 (1.6%)	19 (0.4%)	
5 or more	33 (0.6%)	15 (1.7%)	18 (0.4%)	
Smoking status^c^ (*n* = 2987)				
Never smoked	1796 (60.1%)	262 (55.0%)	1534 (61.1%)	χ^2^ = 75.2 df = 3 *p* < 0.0001
Used to smoke	834 (27.9%)	194 (40.8%)	640 (25.5%)	
Smoke occasionally	142 (4.8%)	7 (1.5%)	135 (5.4%)	
Smoke regularly	215 (7.2%)	13 (2.7%)	202 (8.0%)	
Physical activity^c^ (*n* = 2986)				
Never	918 (30.7%)	213 (44.8%)	705 (28.1%)	χ^2^ = 62.5 df = 5 p < 0.0001
Once a week	571 (19.1%)	86 (18.1%)	485 (19.3%)	
2–3 x per week	869 (29.1%)	95 (20.0%)	774 (30.8%)	
4–6 x per week	459 (15.4%)	45 (9.5%)	414 (16.5%)	
Once a day	121 (4.1%)	26 (5.5%)	95 (3.8%)	
> Once a day	48 (1.6%)	11 (2.3%)	37 (1.5%)	
Occupation (*n* = 5314)				
Employed	3791 (71.3%)	161 (18.8%)	3630 (81.4%)	
Home Duties	375 (7.1%)	40 (4.7%)	335 (7.5%)	
Unemployed	36 (0.7%)	–	36 (0.8%)	
Retired	919 (17.3%)	656 (76.6%)	263 (5.9%)	
Student	193 (3.6%)	–	193 (4.3%)	
Patients referred from^b^:				
GP/FP (*n* = 4217)	280 (6.6%)	64 (9.2%)	216 (6.1%)	χ^2^ = 4.0, df = 1, p = 0.046
Other DC (*n* = 4237)	300 (7.1%)	57 (8.3%)	243 (6.9%)	χ^2^ = 1.5, df = 1, *p* = 0.22
Patient (*n* = 4715)	2421 (51.4%)	366 (51.1%)	2055 (51.4%)	χ^2^ = 0.01, df = 1, *p* = 0.92
Other (*n* = 4200)	917 (21.8%)	124 (18.4%)	793 (22.5%)	χ^2^ = 2.2, df = 1, *p* = 0.14

^a^Pearson χ^2^ test for categorical variables, t-test for means, all accounting for clustering within primary sampling unit of *chiropractor*

^b^Indication of multiple referral sources was possible

^c^Smoking Status and Physical Activity were collected on half of the encounter forms by design

Table 3 presents the characteristics of chiropractic encounters with patients aged < 65 years and ≥ 65 years old. Encounter duration and imaging ordering at encounter were similar between younger and older adult patients. Although some differences can be observed in source of payment between younger and older adult patients, most patients (78% of older adult patients and 77% of younger adult patients) paid out-of-pocket for their chiropractic treatment.

**Table 3:** Patient encounter characteristics for total combined adult encounter sample, for patients aged ≥65 and < 65 years at the encounter level

	Total encounters (*n* = 6781)	Encounters with ≥65 years patients (*n* = 1067)	Encounters with < 65 years patients (*n* = 5714)	Comparison* between ≥65 years and < 65 years
Source				
COAST	3842 (56.7%)	491 (46.0%)	3351 (58.7%)2	χ^2^ = 11.9 df = 1 *p* = 0.0006
O-COAST	2939 (43.3%)	576 (54.0%)	,363 (41.4%)	
Duration of Encounter (minutes)				
Mean (Range; SD; Median)	17.6 (0–120; 11.2)	18.0 (1–120; 11.2)	17.5 (0–120; 11.2)	t = 0.83 df = 90 *p* = 0.41
Imaging ordered/done at encounter				
X-ray spine	196 (2.9%)	38 (3.6%)	158 (2.8%)	χ^2^ = 0.55 df = 1 *p* = 0.46
X-ray other	19 (0.3%)	3 (0.3%)	16 (0.3%)	χ^2^ = 0.0 df = 1 *p* = 0.99
MRI	19 (0.3%)	1 (0.1%)	18 (0.3%)	χ^2^ = 2.12 df = 1 *p* = 0.15
CT Scan	14 (0.2%)	2 (0.2%)	12 (0.2%)	χ^2^ = 0.01 df = 1 *p* = 0.91
Ultrasound	7 (0.1%)	1 (0.1%)	6 (0.1%)	χ^2^ = 0.01 df = 1 p = 0.92
Source of payment				
Workers’ Comp	115 (1.8%)	7 (0.7%)	108 (2.0%)	χ^2^ = 8.09 df = 1 *p* = 0.0045
Auto Insurance	102 (1.6%)	10 (1.0%)	92 (1.7%)	χ^2^ = 0.66 df = 1 *p* = 0.42
Veterans’ Affairs	46 (0.7%)	24 (2.3%)	22 (0.4%)	χ^2^ = 29.81 df = 1 p < 0.0001
Medicare	82 (2.2%)	28 (6.0%)	54 (1.7%)	χ^2^ = 43.88 df = 1 *p* < 0.0001
Private Insurance	2687 (40.8%)	314 (30.4%)	2373 (42.8%)	χ^2^ = 18.11 df = 1 *p* < 0.0001
Patient	5080 (77.2%)	813 (78.7%)	4267 (76.9%)	χ^2^ = 0.52 df = 1 *p* = 0.47
No Charge	229 (3.5%)	32 (3.1%)	197 (3.6%)	χ^2^ = 0.20 df = 1 *p* = 0.66
Other Charge	6 (0.6%)	6 (0.6%)	39 (0.7%)	χ^2^ = 0.32 df = 1 *p* = 0.57

*Pearson χ2 test for categorical variables, t-test for means, all accounting for clustering within primary sampling unit of chiropractor

### Problems diagnosed by chiropractors for patients aged ≥65 years

Table 4 reports the distribution of problems managed (10 most frequent problems) per patient encounter, as reported by the chiropractor. For 92 encounters (1.4% of 6781 encounters), no diagnostic information was provided on the forms. More than half (56%) of the problems managed in older chiropractic patients were back problems, with the next most common being neck problems (10%), radiating back syndrome (5%) and muscle problems (4%). In patients aged < 65 years, the four most common problems managed by the chiropractor were the same, albeit in a different order: back problem (52%), neck problem (14%), muscle problem (5%) and radiating back syndrome (5%). While headache was the 5th most common problem managed in patients aged < 65 years it was not a common problem in older patients. Alternatively, osteoarthritis, which was the 6th most common problem for patients aged ≥65 years, was not a common problem for younger patients.

**Table 4:** Distribution of the ten most frequent problems managed, as reported by chiropractors, for patients aged ≥65 and < 65 years at the patient encounter level

	≥65 years patient encounters (*n* = 1067)				< 65 years patient encounters (*n* = 5714)		
	% of diagnoses (n)	Rate per 100 encounters	95% CI		% of diagnoses (n)	Rate per 100 encounters	95% CI
Back problem	55.7 (863)	82.4	72.7–93.2	Back problem	52.1 (4249)	75.3	67.7–83.8
Neck problem	10.1 (157)	15.0	10.9–20.5	Neck problem	13.7 (1119)	19.8	16.6–23.8
Back syndrome with radiating pain	5.4 (83)	7.9	5.1–12.2	Muscle problem	5.2 (428)	7.6	5.2–11.0
Muscle problem	4.1 (63)	6.0	3.9–9.3	Back syndrome with radiating pain	4.7 (387)	6.9	5.0–9.4
Shoulder problem	4.0 (62)	5.9	4.1–8.6	Headache	2.7 (224)	4.0	3.0–5.3
Osteoarthritis (not spine)	3.8 (59)	5.6	4.0–8.0	Shoulder problem	2.5 (200)	3.6	2.8–4.5
Health maintenance	1.7 (26)	2.5	1.2–5.3	Other musculoskeletal problem	2.3 (191)	3.4	1.6–7.1
Knee problem	1.4 (21)	2.0	1.0–4.1	Health maintenance	2.2 (179)	3.2	1.8–5.7
Foot problem	1.2 (19)	1.81	1.0–3.3	Foot problem	1.6 (130)	2.3	1.5–3.6
Hip problem	1.2 (19)	1.8	1.1–3.1	Knee problem	1.1 (86)	1.5	1.1–2.2

*CI* Confidence interval

### Chiropractic treatment provided to chiropractic patients aged ≥65 years

Soft tissue therapy was the technique most commonly provided to patients ≥65 years being provided in 85 per 100 encounters, and twice as often as mobilization (Table 5). While manipulation was the most common technique provided to patients aged < 65 years (91 per 100 encounters), it was the second most common technique for patients ≥65 years (60 per 100 encounters). Chiropractors reported recommending exercises in 29 per 100 encounters. Mobilization techniques, activator, blocks and other modalities were more commonly provided to patients ≥65 years old than younger patients.

**Table 5:** Techniques and care provided for total combined adult encounter sample, for patients aged ≥65 and < 65 years at the encounter level

	Total encounters (*n* = 6781)*	≥65 years encounters* (*n* = 1067)	< 65 years encounters* (*n* = 5714)
Soft tissue therapy	82.9 (71.7, 95.8)	85.2 (71.3, 101.8)	82.5 (71.3, 95.4)
Manipulation	86.8 (77.8, 96.8)	60.1 (48.9, 73.8)	91.8 (82.6, 102.0)
Activator	33.7 (24.8, 45.9)	43.3 (31.8, 58.9)	31.9 (23.0, 44.4)
Mobilization	26.9 (19.5, 37.3)	42.5 (30.3, 59.5)	24.0 (17.1, 33.8)
Recommended exercise	34.7 (27.2, 44.2)	29.4 (21.8, 39.7)	35.6 (27.8, 45.7)
Drop piece	26.7 (19.9, 35.7)	26.3 (18.6, 37.4)	26.7 (19.8, 36.0)
Other modalities	18.4 (11.9, 28.4)	24.7 (16.2, 37.9)	17.2 (10.8, 27.3)
Blocking	17.0 (10.9, 26.6)	21.4 (13.5, 33.9)	16.2 (10.0, 26.3)
Flexion / Distraction	6.3 (3.0, 13.1)	7.3 (2.9, 18.7)	6.1 (2.9, 12.8)
Acupuncture	5.9 (2.9, 1.9)	5.9 (2.1, 16.5)	5.9 (3.1, 11.4)

*CI* Confidence interval, *SE* standard error

*Rate per 100 encounters 95% CI

## Discussion

This study combined datasets from observational studies in two countries to provide information on for 6781 chiropractor-adult patient encounters. Of these, 16% were aged older than 65 years, higher than that of the COAST study (13%) [25] and lower than the O-COAST study (19%) [20]. In the majority of older patients (60%), the primary problem diagnosed by their chiropractor was a back problem. This is higher than for the total sample across all ages in COAST and O-COAST (50 and 55% respectively) [20, 25]. While we know that the prevalence of back pain in older adults is similar to that in younger people, it is more severe and disabling with increasing age [29]. Whether the severity of back pain is the reason why older adults seek chiropractic care, or it is for more contextual factors (e.g. multisite joint pain, maintenance care or seeking alternatives to surgery) is unclear. The relationship between age, osteoarthritis (spondylosis) and the prevalence of pain is poorly understood. Lee et al. [30], reported that lumbar spondylosis was associated with low back pain among females over 60 years old and that lumbar spondylosis correlated with severity of back pain. However, it is well documented that, in the general population, many radiographic findings show either no or weak association with symptoms [31].

One in seven chiropractic patients (16%) were aged older than 65, which concurs with results (16%) from an earlier Canadian/US practice-based research program [21]. The older chiropractic patient is most commonly female, retired, has previously seen a chiropractor, and paid out of pocket for chiropractic services. One quarter of older patients were aged between 75 and 85 years, and 5% of older patients (and nearly 1% of *all* chiropractic patients) were aged over 85 years, revealing that adults who may be seen as frail also seek chiropractic care. From our study, only 3% of older patients were new patients, suggesting that nearly all older patients had previously visited their chiropractor.

Of the 946 older patients diagnosed with a back problem, 83 (5%) had radiating back pain. Chiropractors report regularly treating patients experiencing low back-related leg pain, and in those chiropractors who ‘often’ treat low back-related leg pain, they were more likely to treat degenerative spine conditions [32]. While the methods of this study do not allow us to report the specific diagnosis of older patients with radiating back pain, symptomatic lumbar spinal stenosis is often characterized by neurogenic claudication, defined as symptoms of pain, weakness and/or numbness radiating into one or both buttock, thigh, or lower leg [33]. In the absence of progressive neurological deficits or cauda equina symptoms, non-surgical approaches are recommended for lumbar spinal stenosis, with conservative care that involves chiropractic (combined with one-on-one instruction on daily exercises and self-management strategies) being superior to self-directed care [34].

In high income countries, such as Australia and Canada, multimorbidity is mainly driven by age, and the proportion of the population living with two or more diseases is steadily increasing because of demographic change. We found that one-fifth of older chiropractic patients had more than one comorbidity, much less than the 75% of patients aged 65–74 years with multimorbidity at Australian general practitioner encounters (which increased to 83% in those aged ≥75 years) [35]. In community-dwelling older women with arthritis, 42% self-reported multimorbidity [12], more than twice the proportion in our study. Differences between doctor-, chiropractor- and self- reported medical conditions, and how they are categorized, may determine the reason for discordance, or it may be that older adults who receive chiropractic care are healthier than those who do not [36]. Treating chronic diseases in isolation leads to complicated and costly interactions within the health system. Unless health systems shift the paradigm to a holistic treatment of the older patient in order to manage the consequences of chronic diseases, an increasing number of older patients may be disadvantaged [37, 38].

Chiropractic management of the older patient most often included soft tissue therapy (57%), followed by spinal manipulation (41%). In a 2015 review by de Luca et al. [39], a limited number of studies (*n* = 5) were found that investigated the effectiveness of manual therapy for chronic low back pain in older adults. Three trials compared different forms of manual therapy, with no significant differences between groups in pain outcomes. All groups had statistically significant improvements in pain over time, suggesting non-specific therapeutic effects of manual therapy. In this study, it was found that mobilization, mechanically assisted adjustment and blocking techniques were more commonly used in older adult patients, likely due to the chiropractor’s perception that these techniques deliver less forces to patients in comparison to manipulation. Indeed, forces used during mobilization techniques and delivered by mechanical hand-held devices (such as activator) have been described to be smaller in magnitude than the ones used during manipulation techniques [40, 41]. It is important to note, however, that while mechanical hand-held devices usually apply similar forces more consistently, forces applied during mobilization techniques can vary depending on the patient and provider. To date, no study has quantified the forces applied during spinal manipulation or mobilization in an older adult population.

While numerous international guidelines suggest exercise as a first-line treatment for back pain [42, 43], in Australia, older adults with back pain who visit a general practitioner are 50% are less likely to be advised about exercise than younger adults [44]. This current study found that in 29 of every 100 encounters, chiropractors recommended exercise to older patients as a part of their treatment. Further exploration of the frequency, intensity and type of exercise recommended by chiropractors, and for what problems exercise was prescribed, is warranted. Low back pain guidelines currently do not recommend exercises differently for older versus younger adults and herein lies a gap in the evidence for the appropriate prescription of exercise to older adults with back pain. Prescription of aerobic, resistance, stability exercises and Tai Chi are recommended for older adults with musculoskeletal conditions such as hand, hip and knee osteoarthritis [45, 46], osteoporosis and osteopenia [47, 48], and headaches associated with neck pain [49]. In addition to prescribed exercise, following guideline advice to stay active will benefit the older adult with musculoskeletal conditions. For example, walking for 30 min on **≥**5 days a week and strength exercises on **≥**2 days per week lower the risk of persistent LBP, after adjusting for age and body mass index [50]. Similarly, strength exercises lower the risk of LBP among men **≥**65 years after accounting for age and BMI [50].

Guidelines recommend that laboratory tests and imaging should not be routinely used as part of early management, and that pharmacological treatment follows only after an inadequate response to first-line nonpharmacological interventions [42, 43, 51]. Unfortunately, health services research in the older patient with low back pain has shown increases in diagnostic studies, injections account for a significant proportion of back pain management costs [52], and in the US, elective spinal fusion surgery in the United States increased by 62.3%, with hospital costs for this procedure exceeding 10 billion USD in 2015. At a health system level, lack of time and training, and limited access to evidenced based information and coordinated health care are barriers to adults receiving guideline recommendations on care for low back pain [53]. Care provided by chiropractors in this study, captured recommendations of first line (exercise) and second line (soft tissue therapy and spinal manipulation) treatments. Furthermore, chiropractors reported x-rays in approximately 4% of patients ≥65 years, a similar proportion as reported in the younger patients. Critical research is needed to determine whether low value care (that is, care that is discordant with international guidelines) is more common in this population, and whether low value care further impacts the physical and psychological health of older adults.

### Strengths

This study combined data from the COAST and O-COAST studies, providing the largest published capture of data from chiropractic practices across two countries. It characterises chiropractic practices that are, from the originally published studies, nationally representative in terms of age, years since graduation and years in practice. Additionally, by combining data from two countries, this study accounted for potential regional or cultural practice characteristics. Chiropractors completed data forms at the time of consultation, minimising recall bias which is an inherent limitation of previous chiropractic workforce studies that rely on practitioner recall [21]. In addition, this study provides clinical information that would not be available in claims data. Finally, coding of clinical information to a specific ICPC-2 PLUS term, the ICPC-2 PLUS Chiro terminology enables standardised grouping of similar concepts (or groups of concepts) for the chiropractic profession [27]. Grouping COAST and O-COAST clinical chiropractic information using an existing and internationally classification terminology has provided data which can be pooled and compared internationally.

### Limitations

Firstly, data from COAST and O-COAST were collected by chiropractors’ and self-reported by patients, which is not necessarily an exact representation of the content of the chiropractic encounters. Validity studies were undertaken by the BEACH (Bettering the Evaluation and Care of Health) study in general medical practice [54], upon which our methods were based. Secondly, descriptions of the reasons for encounter, diagnoses and comorbidities is limited by the ICPC-2 PLUS general practice terminology [26] and the ICPC-2 PLUS Chiro terminology [27]. While the coding of clinical information to a specific term has enabled the standardised grouping of symptoms/complaints, problems/diagnoses or processes of care together, there are multiple uses of non-specific terms such as “back problem and “radiating back syndrome” that do not elucidate specific diagnoses that chiropractors use to accept and treat older patients into clinical practice. Next, low response rates in both the COAST [25] (52 of 156 eligible chiropractors (33%) and O-COAST [20] (42 of 120 eligible chiropractors (35%)) studies, which were higher than the 2011 BEACH study performed in general practice [54], mean that the results may not be generalisable to the broader older adult patient population. Finally, while the COAST and O-COAST sampling method recruited randomly selected chiropractors, and then collected data from consecutive patient encounters, prevalence bias may distort the finding. For example, patients with recurring or persistent symptoms who may be receiving ongoing care would be more likely to be recruited to the study rather than patients presenting with a new complaint.

## Conclusions

From 6781 chiropractor–adult patient encounters across two countries, one in six chiropractic patients were aged ≥65 years. Among older adult patients, back pain was the most common problem diagnosed by chiropractors (accounting for 82 in every 100 encounters). Neck pain and lower limb problems were the next most common presentation to chiropractors. Soft tissue therapy was the most commonly used technique and 29% of older patients were recommended exercise. Among older adults, back pain is the most common problem in chiropractic practice, and future research should explore the clinical course of back pain in older patients seeking chiropractic care.

## Data Availability

This research was completed using data from COAST and O-COAST studies, of which public access is currently open. The datasets used are available from the corresponding author on reasonable request.

## Abbreviations

BEACH: Bettering the Evaluation and Care of Health
COAST: Chiropractic Observation and Analysis Study
HREC: Human research ethics committee
ICPC-2: International Classification of Primary Care
O-COAST: Ontario -Chiropractic Observation and Analysis Study
REB: Research ethics board
STROBE: Strengthening the Reporting of OBservational studies in Epidemiology
